# Optimal Semelparity

**DOI:** 10.1371/journal.pone.0057133

**Published:** 2013-02-19

**Authors:** James W. Vaupel, Trifon I. Missov, C. Jessica E Metcalf

**Affiliations:** 1 Laboratory of Survival and Longevity, Max Planck Institute for Demographic Research, Rostock, Germany; 2 Max-Planck Odense Center on the Biodemography of Aging, University of Southern Denmark, Odense, Denmark; 3 Duke University Population Research Institute, Durham, North Carolina, United States of America; 4 Faculty of Economic and Social Sciences, Institute of Sociology and Demography, University of Rostock, Rostock, Germany; 5 Department of Zoology, University of Oxford, Oxford, United Kingdom; University of Zurich, Switzerland

## Abstract

Semelparous organisms have a simple life cycle characterized by immediate death after reproduction. We assume that semelparous life histories can be separated into a juvenile non-reproductive period followed by an adult period during which reproduction is possible. We derive formulae for the optimal age and size at reproduction and for the optimal size of the offspring (e.g., seeds). Our main contribution is to determine the conditions under which the optimal size of the offspring does not depend on the optimal size at reproduction and *vice versa*.

## Introduction, Assumptions and Notation

“Plants of any size have seeds that vary approximately 400-650-fold between species”, as authors in [1] point out; they note that “*Sequoia sempervirens* has a seed mass of 0.0037 gram.” Animal offspring also vary widely in size. What evolutionary factors determine the size of mature adults vs. the size of their progeny?

This question is the subject of a large body of literature. [2], [3], [4], and [1] provide useful overviews of the literature on plants. An early framework was proposed in [5] and expanded in [6]. [7] developed a different perspective with a focus on mammals. Our contribution is to build a biodemographic framework that unifies predictions about adult size and offspring size in simple, precisely-defined optimization models and to rigorously prove key implications of these models. We achieve simplicity by focusing on semelparous species, which reproduce once and die.

Evolutionary biologists have taken advantage of the simplicity of the semelparous life history. For example, demographic models have been developed to explore how stochasticity affects reproductive delays (see [8]), how variation in growth shapes plasticity in timing of reproduction (see [9]), and how the evolution of reproductive delays interacts with pre-reproductive delays such as seed-banks (see [10]). However, to date, no single analytical framework providing dynamic insights into optimal life-histories of semelparous species has been developed. There is a need for such theory to separate the effects of complexities such as changing predation regimes and resource limitation (see [11]) and stochastic environments (see [8]) from patterns driven by the general principles underlying demographic trajectories. Here we make a start at filling this gap by providing an analytical framework that unifies treatment of the two main axes of life-history variation in such species: the optimal timing of reproduction and the optimal offspring size. We focus on the simplest case of constant environments and constant population size.

The life cycle of semelparous species can be viewed as a two-phase process, driven by different mechanisms. Stage 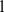 is a juvenile non-reproductive period, in which some individuals survive to become adults. Adults can reproduce and, when they do, they die. Hence stage 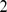 is the period of life in which individuals seek to maximize their reproduction by weighing at each instant the benefits of delaying reproduction further against the risk of death associated with this delay. We assume size 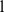 is the milestone between the two phases. Without loss of generality, we can further assume that size 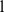 corresponds to adult age 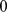. Table 1 summarizes the basic characteristics of stage 1 vs stage 2.

**Table 1:** Life-Cycle Phases for Semelparous Species.

Stage	Growth	Mortality	Reproduction
Stage 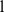	Yes (from 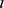 to 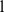 )	Yes	No
Stage 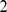	Yes (from 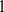 onwards)	Yes	Yes

Let 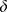 be the duration of stage 1. Let 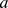 be the age of the organism in stage 2, age 0 in stage 2 being the age when size 1 is reached. Let 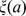, 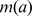, and 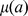 denote at age 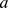 the organism's size, its reproductive capacity, and the force of mortality, respectively. By assumption, 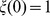. We define reproductive capacity as the expected number of offspring that reach size 1. Let 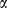 denote the age at which reproduction occurs. Let 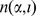 be the number of offspring produced, with each offspring (e.g., seed) being the same size 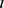. We consider 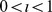. Finally, let 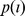, 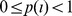, be the probability that an organism born at size 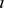 survives to size 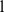. Note that reproductive capacity is given by 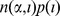.

Using subscripts to denote generations, we define parental size as growing from 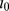 to 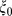 and offspring size as growing from 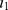 to 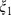.

**Figure 1:**
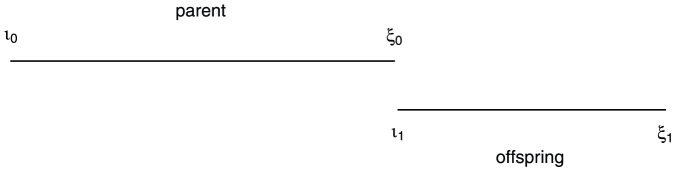
Parent and offspring size notation.

In this article we address three questions about semelparous organisms. First, what is the optimal age (in stage 2) at reproduction and what is the organism's size at this age? Second, what is the optimal number of offspring and what is the optimal size of each offspring? Third and most importantly, does the optimal size of an organism at reproduction 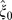 depend on the optimal size of its offspring 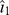 (see Fig. 1)? Our first question is what determines 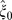, which is assumed to be equal to 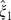. Our second question is what determines 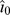 which is assumed to be equal to 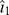. Our third and most important question concerns the relationship between 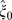 and 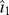. The assumptions we made about the separation of the two stages imply that 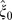 and 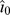 are independent and, similarly, 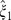 and 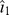 are independent. The question of interest is whether 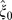 and 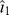 are independent. This formulation has not been clearly developed in previous studies [12] and is a key contribution.

## Semelparous Strategies: Models and Results

### Optimal Age and Size at Reproduction

Stage 2, which starts once seed size no longer affects the risk of dying, is the stage of adult growth during which reproduction is possible. If reproduction occurs only at age 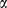 in stage 2, if the chance 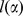 of surviving to 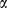 is constant over time and across environments, and if 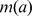 and 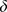 are similarly constant, then the net reproduction rate 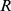 for such semelparous species can be expressed as
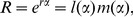
(1)


where 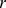 is the rate of population growth, and 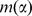 measures reproduction at age 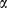; 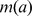 at any age 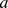 other than 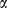 is zero. This implies that
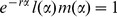
(2)


[13, p189], an expression that follows directly from the Lotka equation,
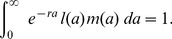
(3)


Proof that 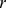 represents the growth rate in the Lotka equation is not straightforward and depends on the assumption of stable populations (see [14]), but (2) for semelparous species is true by definition. The simplicity of (2) facilitates analytical insights into optimal age at reproduction and optimal offspring size.

Solving (2) for 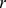 yields (see [13], p.189)
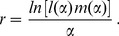
(4)


The value of 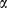 that maximizes 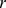 is the optimal age at reproduction, 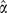. It satisfies the condition
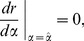
(5)


Inserting the expression for 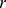 from (4) into (5), using the equation for the derivative to solve for 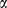, and rearranging terms yields the requirement that the optimal age at reproduction, denoted by 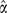, must satisfy:
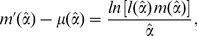
(6)


where 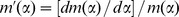 and 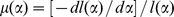. Note that 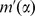 is the relative rate of improvement in reproductive capacity at age 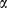, and 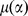 is the hazard of death (force of mortality) at age 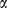. Substituting (4) into (6) shows that
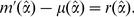
(7)


At equilibrium, 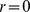 and the optimal age at reproduction is defined by a balance between the rate of growth in reproductive capacity and the force of mortality,
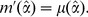
(8)


Note that in reality populations, especially semelparous populations, might not be always at equilibrium. We will, nevertheless, assume they are in order to illustrate the trade-off mechanism in determining the optimal timing of reproduction. From (8), reproduction should be delayed as long as the reproductive benefits of further growth outweigh the risk of mortality occasioned by delaying. The optimal age at reproduction is the age at which the benefits of further growth are exactly offset by the risk of dying. Note that 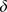, the duration of stage 1, does not appear in (8) and does not affect the optimal age (in stage 2) of reproduction. If the population were growing or shrinking, then 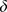 would matter, as it would affect time to reproduction; with earlier times being favored in growing populations (see [15], [16]); and later times in shrinking populations (see [17]). In the rest of this article we focus on the equilibrium case when 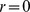 and we will use “age” to refer to age in stage 2.

The optimal size at reproduction 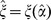 is the size of the semelparous organism at the optimal age at reproduction. We assume semelparous organisms grow until they reproduce, i.e. 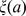 is an increasing function of age (this might not always be the case as shown in [18], [19]). As a result, this optimal size can be determined by
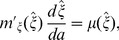
(9)


which results directly from (8) by viewing it as a necessary condition for the optimal size rather than the optimal age. That is, at the optimal size, the increase in reproduction with an increase in size multiplied by the change in size in an additional unit of time (or age) must be counterbalanced by the risk of death during that unit of time.

If environmental conditions worsen such that the rate of growth in reproductive capacity at all ages decreases, when population equilibrium is reached the new optimal 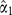 is younger than 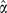. If mortality increases, the optimal age is also younger, 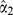. If both occur simultaneously, the optimal age is even younger 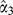.

### Optimal Size at Reproduction in a Specific Model for Stage 2

Both (7) and (9) are true in general, whatever functional forms are used for 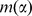 and 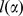. Specific functional forms can be used to make more specific predictions. Mortality is a declining function of size in many species, as larger individuals may be more robust to threats such as droughts, or predation. For example, in semelparous plants, the most commonly observed pattern of mortality is declining with size (12 out of 12 species reviewed in [20]). An appropriate model could therefore be
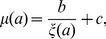
(10)


where 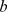 and 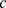 are constants, and 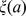 denotes size at age 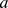. The parameter 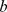 captures the causes of death that decline with size, 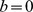 captures no size dependence, and 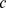 captures ubiquitous causes of death that are independent of size. For many plants, reproductive output scales approximately with biomass, so that allometric relationships can be fitted related seed counts to size (see [20], [21] for a review of estimates for a range of species). As a result reproductive output is generally an increasing function of size and can be modelled as
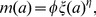
(11)


where 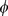 is a scaling parameter and 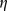 modulates whether transforming size into reproductive output is an accelerating (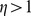) or saturating (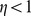) function. In semelparous plants, growth is generally a declining function of size, a function that has been attributed to self-shading, or declining nitrogen content of older leaves (reviewed in [20], [22]). Accordingly, an appropriate model would be
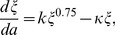
(12)


where the parameter 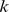 captures how the growth rate increases with size, and 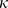 modulates the increase so that eventually size reaches an asymptote. For illustration, we use the exponent 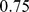, following predictions from the fractal model of scaling (see [23]). However, using a different exponent would not alter the main conclusions of the article. This equation provides a fairly general description of asymptotic growth. If size at age 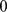 is 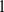, we have
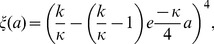
(13)


where the asymptotic size is defined by 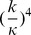.

Substitution of (10), (11), and (12) in (9) results in an expression for the optimal 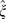 that is explicitly independent of the scaling parameter 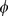


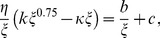
(14)


which reduces to
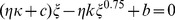
(15)


The latter is a quartic equation for 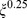 and its analytic solution is given by Ferrari's formula. Denoting
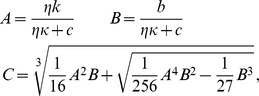



we can express the positive root of the quartic equation (14) in the following manner
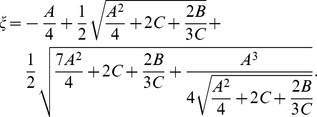



**Figure 2:**
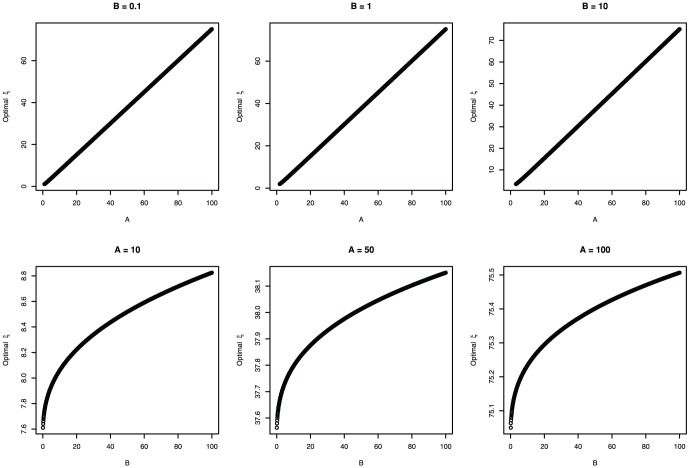
Optimal seed size with respect to A and B.

As a result, 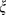 increases with 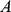 and decreases with 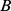 (see Fig. 2). Therefore, the optimal size of reproduction 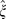 will increase with positive changes in the reproduction scale parameter 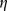 or the determinant of asymptotic size 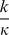, as well as negative changes in mortality parameters 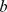 or 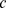.

These mathematical results aid biological insight. Because optimal size does not depend on the parameter 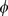, species suffering proportional reduction in offspring production will, certibus paribus, not vary in flowering size (see [24]). An example of this might be density dependence of seed establishment (see [20]). Furthermore, if species' relative ranking with respect to asymptotic size 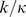, scaling of reproductive output with size 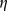, and mortality parameters, 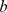 and 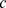, are known, relative ranking in terms of flowering size could be predicted.

### Optimal Seed Size and Number

Let 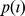 be the probability that a seed germinates and grows until initial size no longer influences mortality, i.e. to 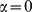 and size taken as 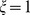. Generally 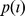 increases with seed size 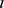. Let reproductive output i.e., number of seeds produced, be denoted by 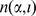 which is an increasing function of plant size (and age), and a decreasing function of seed size. The net reproductive rate is then
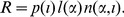
(16)


If the population is in equilibrium, maximizing 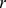 is generally equivalent to maximizing 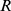 (see [25]). Further, in [24] it has been shown that maximizing 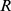 provides the evolutionary stable strategy if population regulation operates on offspring establishment. Such density dependence characterizes many semelparous species (see [20]). The optimal life history is therefore defined by the derivative or relative derivative of 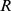 being equal to zero. Hence, the optimal age at reproduction can be specified by
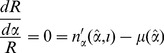
(17)


where 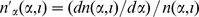 defines the rate of change in the number of offspring produced at age 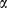. Equation (17) implies 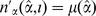, which is similar to the result obtained in (8). Note that optimal time at reproduction depends only on 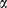 in stage 2 and does not depend on time taken by a seed to grow to 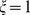 (see [12]). The optimal offspring size is specified by
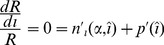
(18)


where 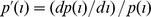 and 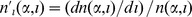. This implies 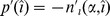. At equilibrium, optimal offspring size is the size at which the benefits accrued through investing less in each offspring and thereby producing more offspring are offset by the risk of mortality for an offspring of that size.

### Optimal Seed Size in a Specific Model for Stage 1

Specific functional forms can be used to deepen understanding. The number of seeds 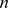 of size 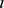 produced at age 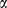 can be determined by
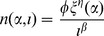
(19)


where parameter 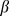 captures both saturating and accelerating functional forms of producing larger offspring. The probability of reaching size 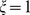 can be specified by a concave function
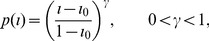
(20)


where 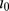 is the minimal possible seed size and 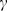 accounts for the speed of reaching reference size 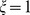. As a result, the optimal offspring size 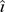 will be the solution of (18) i.e.
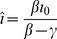
(21)


## Discussion

### When is Optimal Seed Size 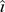 Independent of Optimal Adult Size at Reproduction 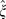


Eq. (21) implies that the optimal seed size 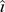 does not depend on the optimal plant size at reproduction 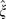. Using (18), it can be similarly shown that optimal plant size at reproduction does not depend on the optimal size of the seeds produced. This mutual independence holds in general if the number of seeds of size 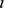 produced at age 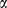 is proportional to the product of a function of adult size and a function of seed size, i.e.
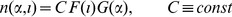
(22)


where 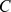 is a scaling factor. In this case
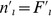
(23)


does not depend on 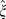 and neither does 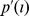. This is also true for
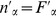
(24)


Eq. (22) is a necessary and sufficient condition, in our framework, for the independence of the parent's optimal size at reproduction from the optimal seed size of its offspring. The condition is not implausible, but it is also not trivial. For instance, in (19) 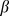 might be a function of 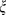: bigger plants might be more efficient at producing large seeds than smaller plants are. Also in (19), 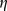 might be a function of 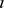: the relationship between plant size and reproductive capacity may be modulated by seed size.

Note that the assumptions about a juvenile vs. an adult stage imply that 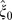 is independent of 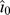 and 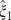 is independent of 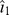 (see Fig. 1). To prove independence of optimal seed size and optimal size at maturity, it is also necessary to show that 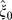 and 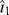 are independent. Eq. (22) gives the condition for this.

The independence of two characteristics means that the optimal value of either of them does not depend on the value of the other characteristic. This causal independence is different from lack of empirical correlation. For instance, suppose a species grows in two environments, one unfavorable (perhaps because of poor soil or lack of sunlight) and the other favorable. Then 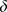, the time it takes a plant to grow from seed to adult size, and 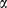, the time it takes for the plant to grow from adult size to size at reproduction and death, might be correlated across the two environments: e.g., both times might be long in the unfavorable environment and short in the favorable one. The long time to develop, however, does not cause the long time to mature: the unfavorable environment causes both and the correlation is merely a statistical association. As explained above, the duration 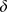 is irrelevant to the optimization problems we addressed.

## Conclusion

The simplicity of the semelparous life cycle aids formulating general mathematical models that predict key features of life histories. The analytical framework presented here unifies predictions of timing of reproduction and offspring size. This framework provides insights into how basic demographic features shape the diversity of age trajectories across species and plasticity within species in response to environmental cues. This permits separation of these patterns from complications such as variation in growth, both across individuals (see [20]) and through time (see [26]). Variants of the models may also be relevant for other life-history switches such as metamorphosis (see [27]).
